# Supplementary figures and images for: Diagnosis of Clostridioides difficile infection by analysis of volatile organic compounds in breath, plasma, and stool: A cross-sectional proof-of-concept study
Source: PLoS One. 2021 Aug 18;16(8):e0256259. doi: 10.1371/journal.pone.0256259 (PMC8372889; doi:10.1371/journal.pone.0256259)

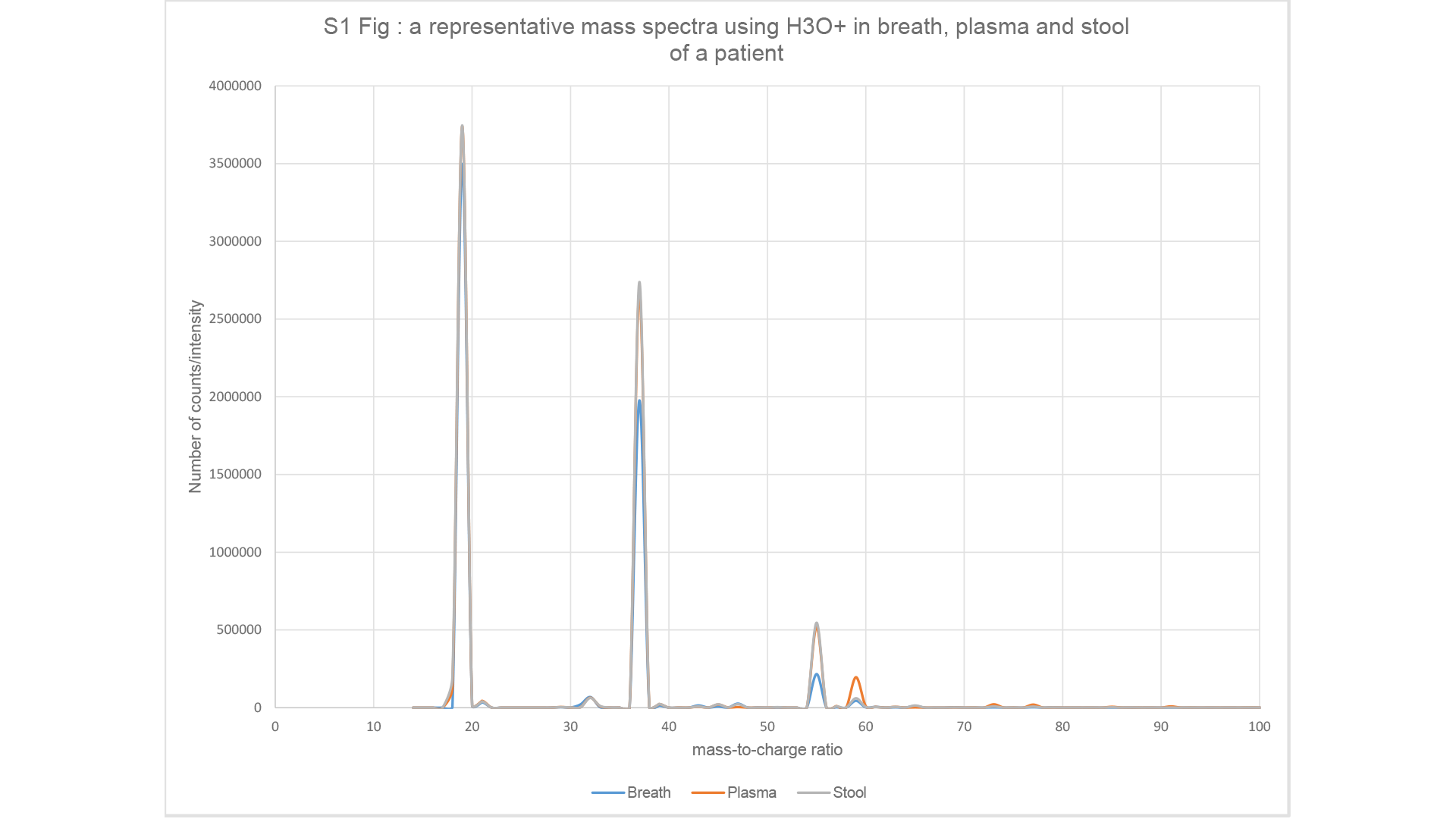

Supplement: S1 Fig — (TIF) [file pone.0256259.s001.tif]

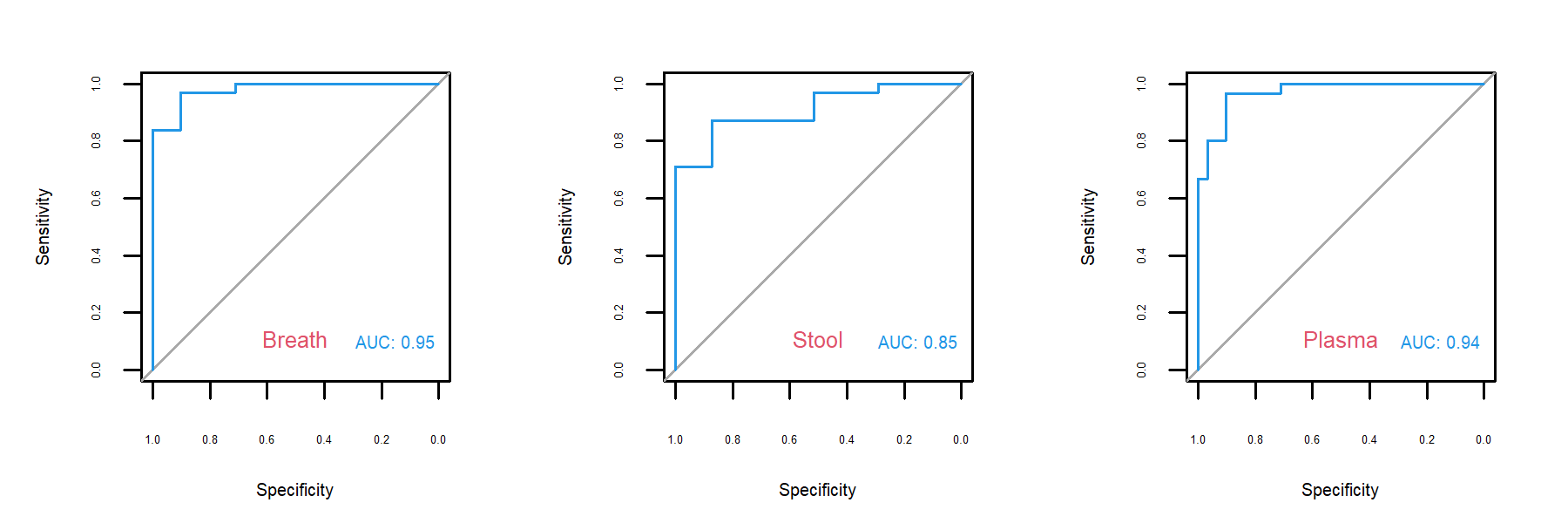

Supplement: S2 Fig — (TIF) [file pone.0256259.s002.tif]

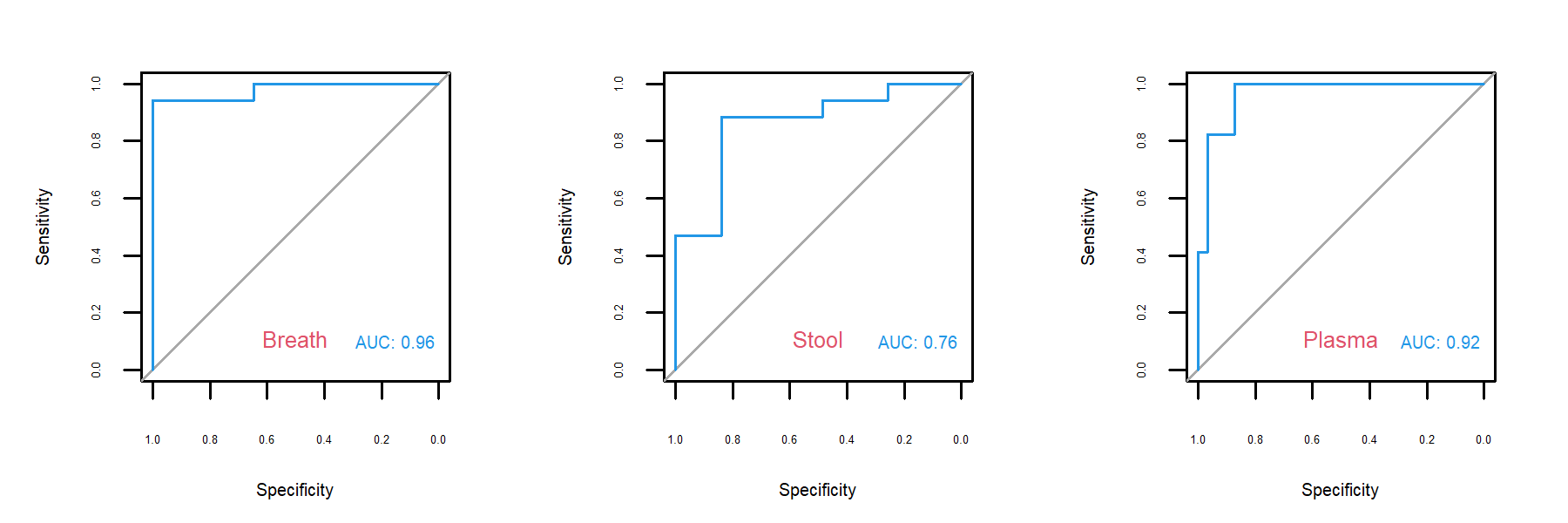

Supplement: S3 Fig — (TIF) [file pone.0256259.s003.tif]
